# Survival analyses of postoperative lung cancer patients: an investigation using Japanese administrative data

**DOI:** 10.1186/2193-1801-3-217

**Published:** 2014-05-01

**Authors:** Susumu Kunisawa, Kazuto Yamashita, Hiroshi Ikai, Tetsuya Otsubo, Yuichi Imanaka

**Affiliations:** Department of Healthcare Economics and Quality Management, Graduate School of Medicine, Kyoto University, Yoshida Konoe-cho, Sakyo-ku, Kyoto City, 606-8501 Kyoto, Japan

**Keywords:** Lung cancer, Survival analysis, Administrative data, Japan

## Abstract

Long-term survival rates of cancer patients represent important information for policymakers and providers, but analyses from voluntary cancer registries in Japan may not reflect the overall situation. In 2003, the Diagnosis Procedure Combination Per-Diem Payment System (DPC/PDPS) for hospital reimbursement was introduced in Japan; more than half of Japan’s acute care beds are currently covered under this system. Administrative data produced under the DPC system include claims data and clinical summaries for each admission. Due to the large amount of data spanning multiple institutions, this database may have applications in providing a more general and inclusive overview of healthcare. Here, we investigate the use of administrative data for analyses of long-term survival in cancer patients. We analyzed postoperative survival in 7,064 patients with primary non-small cell lung cancer admitted to 102 hospitals between April 2008 and March 2013 using DPC data. Survival was defined at the last date of examination or discharge within the study period, and the event was mortality during the same period. Overall survival rates for different cancer stages were calculated using the Kaplan-Meier method. Additionally, survival rates of cancer patients at clinical stage IA were compared between low- and high-volume hospitals using the Log-rank test. Postoperative 5-year survival for patients at stage IA was 85.8% (95% CI = 78.6%–93.0%). High-volume hospitals had higher survival rates than hospitals with lower volume. Our findings using large-scale administrative data were similar to previous clinical registry reports, showing potential applications as a new method in analyzing up-to-date healthcare information.

## Background

Survival rates of cancer patients are a major concern for both patients and physicians, and are often referred to when determining patient prognoses and care strategies.

Cancer registries at the national level support the study of cancer etiology and outcomes, including analyses of survival rates (McLaughlin et al. 2010). In the US, the National Program of Cancer Registries was established in 1992 and provides population-based surveillance. In Europe, the European Network of Cancer Registries (http://www.encr.eu/) and the European Cancer Registry (http://www.eurocare.it/) have been promoting collaborations between cancer registries within Europe and the European Union for more than 20 years. While national cancer registrations are also conducted in Scandinavia and the UK, (Butler et al. 2013) population-based registries in other countries frequently cover only a small portion of the population (Butler et al. 2013). Among these, German registries have progressed to a coverage of 40% in 2012 (Hiripi et al. 2012).

Cancer registrations in Japan are still in the process of development. There exists a population-based cancer registry (http://www.jacr.info/); a hospital-based cancer registry (http://ncc.ctr-info.com/); and “organ-based” registries administrated by various medical associations and organizations, such as the Japanese Joint Committee for Lung Cancer Registration (http://haigan-touroku.jp/). However, these registries possess several shortcomings: for example, the population-based cancer registry has been reported to contain omissions of cases, and survival analyses have not been conducted (Sobue et al.^2007^). The hospital-based registry predominantly includes only data from specialized hospitals, (Sobue et al. 2007). and registries managed by medical associations generally include only voluntary participations. All of these registry databases do not adhere to a single format, and there is no framework that allows for their simple integration (Sobue et al. 2007;Hirata et al. 2012). Furthermore, medical associations conduct and report highly detailed surveys, but tend to struggle with low respondent rates (Sawabata et al. 2011).

The ability to conduct survival analyses using large-scale administrative data, such as healthcare claims databases, would provide valuable information on patient prognoses and treatment effectiveness for a population. In 2003, Japan introduced a hospital reimbursement system known as the Diagnosis Procedure Combination/Per-Diem Payment System (DPC/PDPS). This system is characterized by a requirement for healthcare providers to generate DPC data for each patient per hospitalization for reimbursement purposes. DPC data are uniformly formatted and include not only claims data and procedures, but also summaries of patient clinical information such as principal diagnoses and activities of daily living (ADL); in the case of cancer patients, the data also include disease-specific information, such as whether the cancer is primary or recurrent and the TNM classification of malignant tumors. In this way, the DPC/PDPS is in effect a registry of sorts for cancer patients who receive treatment at these hospitals.

Relatively short-term indicators for acute diseases, such as 30-day mortality and in-hospital mortality, have been widely studied (Lee et al. 2013;Sasaki et al. 2013;Yamashita et al. 2013) and applied as quality indicators (The Joint Commission 2014). Long-term survival may also be considered as a possible measure to describe the quality of healthcare. Although it should be kept in mind that long-term survival can be influenced by various determinants including environmental factors, differences in patient survival rates among institutions and regions are a common concern and have been studied (Meyerhardt et al. 2003;Hebert-Croteau et al. 2005).

Research using administrative data may allow the investigation of hospitals that do not participate in any cancer registry as well as hospitals across the boundaries of existing registries. The majority of acute care hospitals in Japan are reimbursed under the DPC system: over 1,400 hospitals are managed under this system as of 2013, and constitute more than 50% of all hospital beds in Japan. The clinical information available in these databases has allowed researchers in Japan to analyze medical care at the national, regional, hospital, and individual levels. With the increasing use of such administrative data for analyses, we are now able to shed light on relatively small hospitals that have potentially played a considerably large role in Japanese healthcare, but have heretofore been unavailable for analysis.

In 1995, our department established the Quality Indicator/Improvement Project (QIP; http://med-econ.umin.ac.jp/QIP/) in order to improve quality of healthcare in participant hospitals through the development, analysis, and feedback of quality indicators. Hospitals voluntarily participate in this project, and there are more than 400 hospitals currently enrolled. These participant hospitals continuously provide DPC data to the QIP; this data is analyzed and the results are periodically reported in feedback to participant hospitals. As this project has involved the collection of data for a relatively long period, it has developed the capacity for long-term analyses.

In this study, we attempt to open a new vista to conduct survival analyses of cancer patients using administrative data, focusing our analysis on postoperative lung cancer patients. This sample was selected due to the high incidence of the disease and high mortality rate of the patients, (Ministry of Health, Labour and Welfare 2013) thereby making this one of the most important fields in health services research. Our analysis may reveal up-to-date details of patient survival with relatively high external validity, as our sample includes hospitals not currently enrolled in any cancer registry. Additionally, we investigate if there are differences in long-term survival among hospitals according to patient volume; the volume-outcomes relationship has been demonstrated in previous studies, and we analyze if our database and methodology are able to obtain similar conclusions to these registry-based studies.

## Methods

### Data

**Figure 1:**
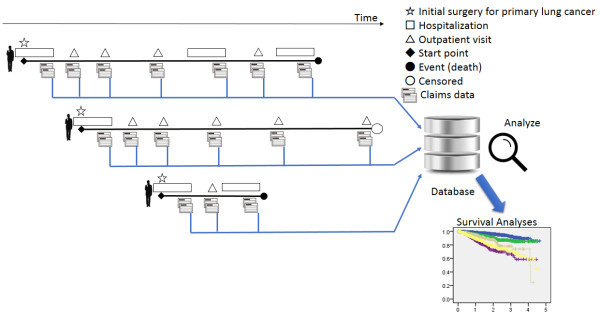
**Data collection and analyses.** All medical claims data produced for each hospitalization and subsequent outpatient visits are collected into a database, which is then analyzed in order to detect and trace statuses and progress of individual research subjects.

In this retrospective cohort study, we analyzed the postoperative survival of non-small cell lung cancer patients using administrative data. The data were obtained from the DPC-formatted database administered by the QIP. This database is very different from those of clinical registries: registries usually collect specific data for predetermined purposes and these data are submitted intentionally for analysis. In contrast, the DPC database uses medical claims data, which are routinely produced for all medical services with the primary intended purpose of reimbursement. These claims data are collected and analyzed to detect the statuses and progress of patients (Figure 1).

Non-small cell lung cancer patients were identified using International Classification of Diseases, 10th revision (ICD-10) codes C34$ combined with cancer-specific information from the DPC data. Patients were included in analysis if they had been discharged from QIP member hospitals between April 2008 and March 2013 with the complete data necessary for our study. Although DPC data are produced either for inpatients or for both inpatients and outpatients depending on hospital, we selected hospitals that had contributed both inpatient and outpatient data to the database.

The details of operations for non-small cell lung cancer were identified from inpatient data. We selected patients who had undergone an operation for the first time for primary non-small cell lung cancer. We investigated patient-level treatment histories from both the inpatient and outpatient database, and identified the last date of medical care or examination for each patient. Survival time was defined as the duration from the date of the initial operation for non-small cell lung cancer to the last recorded date for any medical service provided in the same hospital. For the survival analysis, the event was defined as patients who had died on the last recorded date for medical services, and surviving patients at the last recorded date were regarded as right censored.

It should be noted that the treatment histories for a single patient could only be followed for treatments provided in the same hospital. Therefore, censored cases include cases lost to follow-up due to changes in hospital, in addition to censoring resulting from the end of the study period. For this study, we assumed that the majority of patients would obtain healthcare from the same hospital after operation, as follow-up care would likely be conducted by specialists who are familiar with the patients.

Data concerning the base characteristics of patients and clinical TNM classification of cancer were also analyzed. We reclassified the TNM classes according to overall stage grouping. Because the study period included a transitional period with regard to the shift from Union for International Cancer Control (UICC)-6 to UICC-7 classification, our analysis included an overlap of both types of classification. We identified and standardized equivalent stages from the different staging standards for analysis: for example, T2N0M0 was equivalent to Stage IB in UICC-6; but under the current UICC-7 classification, Stage IB is equivalent to T2aN0M0 and Stage IIA is equivalent to T2bN0M0. Thus, a classification of Stage IB in this study includes both T2N0M0 of UICC-6 classification and T2aN0M0 of UICC-7 classification. We selected patients at Stages IA–IIIA for analysis.

### Comparison of survival rates according to patient volume

In addition, we compared survival rates between high-volume and low-volume hospitals. The relationship between patient volume and quality of healthcare has been widely reported, but there remains a lack of consensus on the nature of the relationship (XMerlino 2007;Luchtenborg et al. 2013;Howington et al. 2013). We divided hospitals into two groups: those that had performed 30 or more operations per year, and those with fewer than 29 operations per year. For ease of comparison, we limited this analysis to patients with non-small cell lung cancer of stage IA (T1N0M0 in both UICC-6 and UICC-7) and who were completely independent with regard to ADL (i.e., patients with a score of 100 points in the Barthel Index).

### Statistical analysis

Overall survival time for the various cancer stages was calculated using the Kaplan-Meier estimator with 95% confidence intervals (CIs).

To analyze the effect of patient volume on the survival of postoperative stage IA lung cancer patients, we utilized the Log-rank test. All statistical analyses were conducted using SPSS software, Version 20.0.0.2 (IBM Inc., Japan).

### Ethical standard

Prior to the study, the study procedures were reviewed and approved (#E553) by the ethics review committee of Kyoto University Graduate School of Medicine, and informed consent was waived. The study complied with the Ethical Guidelines for Epidemiological Research of the Japanese national government, which include guidelines on protecting patient anonymity, and all the necessary conditions were satisfied for informed consent to be waived.

## Results and discussion

### Results

#### Survival rates of postoperative non-small cell lung cancer patients

**Figure 2:**
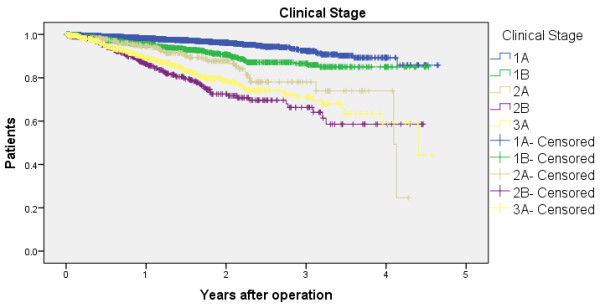
**Survival curves according to clinical cancer stage.** Survival curves according to clinical cancer stage for surgically managed primary non-small cell lung cancer patients.

We analyzed 7,064 primary non-small cell lung cancer patients from 102 hospitals. The various survival rates (1 year to 5 years) in stage IA to IIIA patients are presented in Table 1. The postoperative 5-year survival rate for stage IA patients was 85.8% (95% CI = 78.6% - 93.0%). There were substantially more cases with Stages IA and IB when compared with the other stages. For comparative purposes, Table 1 also shows survival rates from previous reports by the Japanese Joint Committee for Lung Cancer Registration (JJCLCR) in 2011 (Sawabata et al. 2011) and the International Association for the Study of Lung Cancer (IASLC) in 2009 (Tanoue and Detterbeck 2009). Figure 2 shows the Kaplan-Meier curves for overall survival.

**Table 1:** **Clinical stage–specific survival rates (1 year to 5 years) of postoperative non-small cell lung cancer patients**

		Survival rates (%) and number of subjects at risk													
	N	1Y			2Y			3Y			4Y			5Y	
Clinical stages (DPC data)		%	95% CI	n^a^	%	95% CI	n	%	95% CI	n	%	95% CI	n	%	95% CI
IA	3812	98.2	(±0.6)	2014	96.3	(±0.8)	939	92.6	(±1.8)	318	89.3	(±3.1)	36	85.8	(±7.3)
IB	1581	95.4	(±1.2)	781	90.7	(±2.2)	365	86.6	(±2.9)	137	85.0	(±3.7)	25	85.0	(±3.7)
IIA	420	94.6	(±2.5)	200	87.8	(±4.7)	84	78.0	(±8.0)	21	73.9	(±11.0)	4	24.6	(±39.6)
IIB	480	86.4	(±3.7)	230	71.7	(±5.9)	92	66.3	(±7.6)	34	58.6	(±10.6)	7	58.6	(±10.6)
IIIA	771	89.1	(±2.5)	386	78.7	(±4.1)	146	71.1	(±6.1)	58	59.3	(±11.6)	12	44.5	(±26.7)
JJCLCR (2011) Clinical stages 6th edition^b^															
IA	6295	97.0			92.7			89.1			85.5			82.0	
IB	2788	91.0			81.9			74.8			68.0			63.4	
IIA	203	89.7			75.0			64.3			59.2			55.4	
IIB	899	83.7			69.5			59.8			54.0			48.6	
IIIA	940	80.9			64.3			53.6			47.7			43.3	
JJCLCR (2011) Clinical stages 7th edition^b^															
IA	6295	97.0			92.7			89.1			85.5			82.0	
IB	2339	92.5			84.4			77.6			70.8			66.1	
IIA	819	88.7			85.4			66.8			60.2			54.5	
IIB	648	80.0			63.6			54.7			50.4			46.4	
IIIA	1216	81.4			64.7			53.7			47.3			42.8	
IASLC (2009) Clinical stages 6th edition^c^															
IA	831													50	
IB	1842													40	
IIA	25													24	
IIB	2151													25	
IIIA	3005													18	
IASLC (2009) Clinical stages 7th edition^c^															
IA	831													50	
IB	1284													43	
IIA	483													36	
IIB	2248													25	
IIIA	3175													19	

^a^Number at risk.

^b^Data from the Japanese Joint Committee for Lung Cancer Registration (JJCLCR) in 2011 (Sawabata et al. 2011); 95% CI were not available for these data.

^c^Data from the International Association for the Study of Lung Cancer (IASLC) in 2009 (Tanoue and Detterbeck 2009); 95% CI were not available for these data.

#### Comparison of survival rates according to patient volume

**Figure 3:**
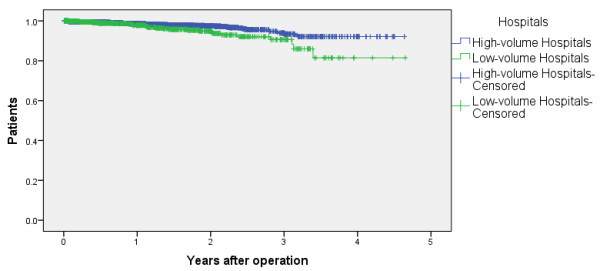
**Survival and patient volume per hospital.** Survival curves by patient volume per hospital for surgically managed primary non-small cell lung cancer patients with a clinical stage of IA.

For the analysis of survival according to patient volume, the sample consisted of 3,379 patients at clinical stage IA with complete independence in terms of ADL. The high-volume hospital group comprised 33 hospitals with 2,587 cases, and the low-volume hospital group comprised 63 hospitals with 792 cases. The base characteristics of these patients are shown in Table 2, and Chi-square tests revealed no significant differences (P > 0.05) in gender and ages between the two hospital groups. The effect of patient volume on survival was assessed using the Log-rank test for the Kaplan-Meier survival curves. Table 3 and Figure 3 show the survival rates of each group. The 5-year survival rates for high-volume hospitals and low-volume hospitals were 92.1 ± 2.9% and 81.4 ± 11.1%, respectively; the results showed a significant difference between the groups (p = 0.05).

**Table 2:** **Base characteristics of patients admitted to low-volume hospitals (n = 63) and high-volume hospitals (n = 33)**

		Low-volume hospitals	High-volume hospitals
Gender	Male	467	1463
	Female	325	1124
Age	<65 y	246	857
	65–74 y	288	986
	≥75 y	258	744
Death		29	52
Total		792	2587

Values in the table indicate numbers of patients.

**Table 3:** **Survival in surgically managed primary non-small cell lung cancer patients with a clinical stage of IA categorized by patient volume per hospital**

		Survival rates (%) and number of subjects at risk													
	N	1Y			2Y			3Y			4Y			5Y	
Patient Volume		%	95% CI	n^a^	%	95% CI	n^a^	%	95% CI	n^a^	%	95% CI	n^a^	%	95% CI
High-volume Hospitals	2587	98.5	(±0.6)	1336	97.3	(±0.5)	614	93.9	(±1.1)	195	92.1	(±2.9)	3	92.1	(±2.9)
Low-volume Hospitals	792	97.8	(±1.2)	427	94.9	(±2.4)	163	90.6	(±4.5)	50	81.4	(±11.1)	18	81.4	(±11.1)

^a^Number at risk.

### Discussion

In this study, we conducted postoperative survival analyses of lung cancer patients using an administrative database. As shown in Figure 2, survival rates varied substantially among the clinical stages. The findings were similar to those in previous Japanese reports that utilized registry data, (Sawabata et al. 2011) which supports the applicability of our administrative data–based method. The second analyses showed that our findings corroborate the results from previous studies in which higher volume hospitals showed a higher survival rate.

The contribution of this study to the field lies in the use of large-scale administrative data to analyze long-term postoperative survival. The comparably larger sample sizes for Stages IA and IB strengthen the validity of our findings; however, the sample sizes were smaller in later-stage cancer, and the details of survival rates for these groups were more difficult to discern. The survival rates for the two early stages were very similar to those previously reported in Japan, (Sawabata et al. 2011) which supports the applicability of this method, although it should be noted that the figures were very different from those in an international report (Tanoue and Detterbeck 2009). Survival rates in this study were slightly higher than those previously reported in Japan (Tanoue and Detterbeck 2009). This discrepancy may be the result of a possible bias in our study due to the loss to follow-up of patients who had died in a different hospital from the initial hospital where the operation was conducted, which is discussed in further detail below. Alternatively, the differences in results may be due to the differences in composition of the study samples, wherein our analysis may have contained more general hospitals than analyses using only hospitals enrolled in cancer registries. Also, the differences in study periods between our analysis and previous studies may have contributed to the observed differences in survival rates. Our study comprises up-to-date data, and may have benefited from general progress in medical care. Further studies are needed to clarify these differences.

The data used in our analysis were only obtained from the administrative data submitted by each hospital, and were not merged with external data sources such as population databases or registration databases. Database integration in Japan presents considerable difficulties due to a lack of national unique identification data, such as social security numbers or national insurance numbers. DPC data may represent a tool to overcome these difficulties, as hospitals under the DPC/PDPS encompass more than half of all acute care beds in Japan, and treat approximately 90% of all acute inpatients (Murata et al. 2013). As DPC data are uniformly formatted, quick multi-institutional analyses are possible and can provide valuable up-to-date information for policymakers, healthcare providers, and the general public.

Hospitals with different patient volumes were found to have different survival rates in our study, which was compatible with previous reports wherein higher volume hospitals showed more favorable outcomes (Merlino 2007;Luchtenborg et al. 2013;Howington et al. 2013). Furthermore, as DPC data contain some clinical information, risk adjustment is possible. Owing to prompt analysis, the use of these data may allow long-term survival to be established as a quality indicator, with applications in comparing regional variations or identifying hospitals with exceptionally low survival rates.

Another point worth noting is that this method can be adapted to other patient populations, such as for other cancers and other diseases. Furthermore, this type of study may have applications in countries other than Japan. Japanese DPC data may serve as a model when designing or reforming other insurance claims databases, as this system not only enables analyses of in-hospital situations, but also provides useful medical data for analyzing patients after being discharged.

Another novelty of this study is that owing to the extensive database, we were able to deal with relatively large sample sizes for each analysis. The benefits of large-scale analysis were demonstrated in the narrow 95% CIs; even the CIs around 5-year survival—which was the full time period of our study—were considerably narrow, indicating a level of reliability in our findings. In other words, we were able to fully utilize the inpatient data spanning a 5-year period by evaluating 5-year survival rates. This was largely due to the use of the Kaplan-Meier method. When the sample size is relatively small, researchers need to wait for many years to obtain a sufficient sample size for an analysis: for example, several years may be required to enroll sufficient cases and several more years to conduct observations after the last enrollment, leading to a lengthy data collection period. However, the DPC data allow quick and up-to-date insight into patient survival over several years, which can support medical staff and policymakers in monitoring current cancer treatments and determining appropriate responses. For example, comparisons of survival rates among different regions may indicate a necessity to re-formulate current healthcare plans.

A limitation of this study is that the administrative data do not include extensive clinical information. For example, DPC data do not contain information regarding pathological TNM stages and pathological types, which are provided in current cancer registries’ reports. Other clinical factors such as comorbidities and co-treatments may influence patient survival, and their effects should also be investigated in future analyses. Therefore, we are unable to make definitive conclusions regarding the effects of hospital case volumes on survival without accounting for such factors. However, our results were compatible with those from previous reports. As our method provides the ability to rapidly assess overall situations, DPC data collection criteria can be expanded in the future to include more clinical information if deemed necessary for analysis. This would, however, require the need to weigh the time and effort in implementing additional data collection against its benefits.

Next, the analyses were limited to tracking data from one hospital for each patient. The starting date for analysis of each patient was the initial surgery date for lung cancer at a DPC hospital. Therefore, there may be time lapses between the initial diagnosis of cancer and the initial treatment. We were unable to determine if a patient had obtained treatment at other hospitals prior to our analysis, as the data could not be interlinked among the various institutions. However, the vast majority of treatment for cancer would likely be conducted in specialized medical institutions, and our method therefore still has validity in investigating cancer epidemiology in Japan. Similarly, the data only allow the tracking of patient care within the same hospital, and patients who subsequently seek healthcare at other institutions are lost to follow-up. Thus, the survival rates reported in this study may be higher than the actual survival rates, as some patients may have sought healthcare or died in other hospitals or hospices. We, at this time, assume that most postoperative patients for lung cancer are followed up at the same hospital where they had undergone surgery, and the majority of recurrent cases or patients with deteriorating conditions are likely to be treated at the same hospital. In addition, some patients may travel a relatively long distance from their homes to undergo specialized surgery, but receive follow-up care at a closer regional hospital. As such cases would also be lost to follow-up, future studies that focus on patients residing in the same region as the hospital where they receive care may reduce this loss of data.

It should be emphasized that there is a need for databases composed of public data to be systematically designed with the intrinsic capability to integrate with other databases in order to maximize their contributions and reduce loss to follow-up. The limitations of the data source used in this study arose from the lack of integration across Japanese public databases. The importance of this study lies in the finding that routinely collected medical claims data have applications in long-term survival analyses. While registry databases are undeniably useful, the integration of other large databases, such as those comprising claims data, should be considered in the analysis of any healthcare topic. Although further studies are needed to improve the follow-up ability of the data and reduce possible bias due to the aforementioned limitations, the integration of data sources is an essential step for improving health services research. In order to both strengthen the validity of analyses similar to this study and reinforce registry databases, public database systems should be designed to allow for integration with other databases. We believe that the further discovery and validation of effective applications of administrative claims databases would support the provision of up-to-date information to help improve healthcare quality and outcomes.

## Conclusions

We conducted survival analyses of postoperative non-small cell lung cancer patients using a Japanese administrative claims database, which covered a wide range of hospitals that included providers not enrolled in any cancer registry. Our findings were consistent with previous reports using analyses of registries, showing that this methodology may have applications as a useful outcomes measurement tool for medical staff and policymakers.

## Abbreviations

(DPC/PDPS): Diagnosis Procedure Combination Per-Diem Payment System
(ADL): Activities of daily living
(ICD-10): International Classification of Diseases, 10th revision
(QIP): Quality Indicator/Improvement Project
(UICC): Union for International Cancer Control
(CI): Confidence interval
(JJCLCR): Japanese Joint Committee for Lung Cancer Registration
(IASLC): International Association for the Study of Lung Cancer
